# Exercise-mode-related changes in task-switching performance in the elderly

**DOI:** 10.3389/fnbeh.2015.00056

**Published:** 2015-03-06

**Authors:** Chia-Liang Tsai, Wen-Liang Wang

**Affiliations:** Lab of Cognitive Neurophysiology, Institute of Physical Education, Health and Leisure Studies, National Cheng Kung UniversityTainan, Taiwan

**Keywords:** open-skill, closed-skill, cognition, aging, exercise mode, task-switch

## Abstract

The objective of the current study was to explore the relationships between exercise modes and executive functions in the elderly. Twenty-one elderly individuals in the open-skill group, 22 in the closed-skill group, and 21 in the sedentary-behavior (control) group were recruited in the current study, and performed a task-switching paradigm during which the switches occurred unpredictably and infrequently, while the behavioral and electrophysiological performances were assessed simultaneously. The results indicated that although there were no group differences in accuracy rates, the two exercise groups exhibited shorter reaction times (RTs), and larger P2 and P3 amplitudes across all conditions compared to the control group. In addition, the exercise-mode differences revealed a relatively smaller specific cost, and faster motor RTs and larger P3 amplitudes, in the switch condition for the open-skill group in comparison with the closed-skill and control groups. These findings suggest that regularly participating in physical exercise can enhance behavioral and electrophysiological performance with regard to executive control in the elderly, and provide further evidence for the beneficial effects of open-skill exercise on the task-switching paradigm.

## Introduction

Human life spans are increasing due to medical and technological advances. However, due to age-related declines in the volume of cerebral white matter (Sexton et al., 2014), as well as decreases in the concentration, synthesis and number of receptor sites for neurotransmitters (Chen, 2014), a broad array of cognitive functions tend to deteriorate with age (Hedden and Gabrieli, 2004). Indeed, cognitive declines among people aged 65 or older are now a pressing health care issue in many countries. If we cannot find effective primary prevention strategies to ward off the onset or progression of cognitive declines among seniors, the rapidly increasing population of elderly people will be associated with an increase in the number of individuals suffering from mild cognitive impairment, or even dementia (Pressley et al., 2003). Fortunately, numerous large prospective cohort studies have demonstrated that habitual physical exercise could attenuate the cognitive declines that often accompany aging (Colcombe et al., 2004; Wang et al., 2014; Tsai et al., 2015).

There is a growing body of literature which finds that physical activity or exercise can have the positive effects on the brain with regard to neural functioning and cognitive performance (Cassilhas et al., 2007; Smith et al., 2013), and these can be attributed to the processes of neurogenesis, vascularization, and increased blood flow in the brain (van Praag et al., 1999; Endres et al., 2003; Pereira et al., 2007), as well as changes in the secreted levels of some biomarkers in the neurochemical system (Neeper et al., 1995; Tsai et al., 2014b,c). However, there are a broad range of physical exercise modes, and this study roughly categorized them into two main types—open- and closed-skill (Di Russo et al., 2010; Dai et al., 2013). Although both exercise modes have been reported as potentially useful with regard to enhancing neuropsychological and neurophysiological performances (Tsai, 2009; Di Russo et al., 2010; Tsai et al., 2012, 2014a; Dai et al., 2013), the findings in the rather limited research literature remain rather ambiguous (Di Russo et al., 2010; Dai et al., 2013; Wang et al., 2013a,b), and thus these issues need to be further explored.

Individuals participating in open-skill types of exercise (e.g., football, table tennis, basketball, and badminton) have to continually adapt their movements and switch strategies to fit the constantly changing environment and the various skill levels of the other players. Their skills are thus predominantly externally-paced and perceptual (Di Russo et al., 2010). In contrast, the individuals participating in closed-skill types of exercise (e.g., swimming and running) are in a stable and predictable environment, and can perform the exercise according to their own pace. Therefore, their skills tend to be habitual and self-paced (Di Russo et al., 2010). For these reasons, participation in open- and closed-skill exercise requires different sets of motor skills (e.g., initiating appropriate actions, inhibiting inappropriate actions, and switching from an intended movement to another one which is a more suitable response to the opponent’s actions) and different cognitive and executive process loads (e.g., such as planning, selecting relevant sensory information, obeying rules, and cognitive flexibility) (Di Russo et al., 2010). The fact that elderly people with higher levels of cardiorespiratory fitness and physical activity show better cognitive performance is well documented in the vast majority of related research (Hillman et al., 2006; Themanson et al., 2006; Netz et al., 2011; Frederiksen et al., 2014), and, compared to other aspects of cognitive functioning, executive functions are more strongly affected by exercise or physical activity (Etnier et al., 1997; Colcombe and Kramer, 2003). However, no data are available with regard to the effects of different exercise modes on executive control in the elderly with similar cardiorespiratory fitness levels and participating in open- or closed-skill exercise. The main purpose of this study was thus to explore the relationship between exercise type and executive function in the elderly.

The task-switch paradigm involves stimulus perception and identification, task-set updating, attentional reallocation, and response conflict detection and monitoring processing (Friedman et al., 2008). This cognitive task was thus adopted in the current study, since such executive functioning is required and relevant to the changing environment of the open-skill exercise. In general, the task-switch paradigm has been demonstrated to evoke the different visual response components with regard to the resulting event-related potentials (ERP), such as P2 to the target for a unique component of the switch cost or cognitive control on task-set activation (Cepeda et al., 2001; Kieffaber and Hetrick, 2005; Periáñez and Barceló, 2009), and P3 for the collection of processes subsumed under the construct of the task-set reconfiguration (Kieffaber and Hetrick, 2005; Nicholson et al., 2005). There have been previous reports of age- and physical-activity-related effects in these two ERP components in the task-switching paradigm among the elderly (Hillman et al., 2006; Friedman et al., 2008; Adrover-Roig and Barceló, 2010). Therefore, these two ERP components were used in the current study to better understand the effects of different exercise modes on electrophysiological performance in the elderly.

Recently, although Dai et al. (2013) attempted to explore the effects of different exercise modes on executive function using a task-switching paradigm with predictable and frequent switches in elderly subjects who regularly participated in either open- (e.g., table tennis or tennis) or closed-skill (e.g., jogging or swimming) exercise in the previous 3 months, they found that similar exercise effects were observed in the P3 amplitude and P3 latency, and in the reaction times (RTs) of both the global and local switch costs, between the open- and closed-skill groups. The results could be attributed to simple and well-established psychological mechanisms elicited by such a task-switching paradigm with repetition priming, during which participants could prepare the new task set in advance, which resulted in reducing the top-down, reconfiguration process (Friedman et al., 2008). van Asselen and Ridderinkhof (2000) observed age-related increases in specific switch costs when switches are infrequent (20% occurrence) and unpredictable. We thus used a cognitive task during which the switches occurred unpredictably and infrequently, and cues signaling a switch were presented at the same time as the target digits, as this is presumed to require more cognitive processing load. Since switches are unpredictable and/or occur infrequently in the open-skilled exercise situation, and the elderly subjects participating in such an exercise mode should have an advantage over those in the closed-skill one in the task-switching paradigm, we hypothesized that the subjects with long-term experience (i.e., at least 2 years) of open-skill exercise would show better switch-related neuropsychological and electrophysiological performances than those with only closed-skill experience.

## Methods

### Participants

Sixty-four older adults aged between 60–77 years participated in this study: 21 (seven females) in the open-skill group, 22 (eight females) in the closed-skill group, and 21 (eight females) in the sedentary-behavior (control) group. Participants in the open- and closed-skill groups separately participated in either open- (e.g., badminton or table tennis) or closed-skill exercises (e.g., jogging or swimming) at least three times per week for 30 min per session in the previous 24 months. Their demographic characteristics are presented in Table 1. All participants reported being free of a history of brain injury, cardiovascular or metabolic disease, neurological disorders, any medications that influence central nervous system functioning, and had normal (or corrected to normal) vision based on the minimal 20/20 standard. None of the participants showed any symptoms of psychiatric illnesses, such as depressive symptoms (all scored below 13 on the Beck Depression Inventory, 2nd edition (BDI-II, Beck et al., 1996)), dementia, or mild cognitive impairment (all scored above 26 on the Mini-Mental State Examination (MMSE, Folstein et al., 1975)). Written informed consent, as approved by the Institutional Ethics Committee of National Cheng Kung University, was provided by all the subjects, who were paid NT $1500 for their participation.

**Table 1:** **Demographic characteristics of the open-skill, closed-skill, and sedentary-behavior (control) groups**.

	Open-skill (*n* = 21)	Closed-skill (*n* = 22)	Control (*n* = 21)	*p*
Age (years)	65.35 ± 4.21	66.03 ± 4.07	63.94 ± 3.36	0.211
Gender (female)	7	8	8	0.949
Height (cm)	164.32 ± 7.11	163.87 ± 7.50	163.94 ± 7.71	0.977
Weight (kg)	64.13 ± 10.48	62.75 ± 7.99	64.21 ± 8.98	0.841
Body Mass Index (kg/m^2^)	23.68 ± 3.16	23.40 ± 2.82	23.85 ± 2.75	0.874
Education (years)	13.71 ± 2.95	13.45 ± 3.49	12.86 ± 2.03	0.619
Systolic pressure (mmHg)	122.43 ± 23.35	126.95 ± 15.85	122.29 ± 26.45	0.736
Diastolic pressure (mmHg)	76.62 ± 12.19	80.64 ± 13.58	77.29 ± 11.76	0.533
Hypertension (male)	3	4	3	0.921
Hypertension (female)	2	1	0	0.350
MMSE	27.76 ± 1.30	28.23 ± 1.11	27.29 ± 1.52	0.072
BDI-II	3.29 ± 3.35	2.95 ± 3.27	5.00 ± 3.82	0.130
Social participation	9.33 ± 2.56	10.23 ± 1.97	10.76 ± 2.45	0.143
VO_2max_ (mL/kg/min)	30.67 ± 6.56	32.81 ± 4.90	25.36 ± 4.66	<0.001^*^
Memory depth	21.10 ± 4.19	21.55 ± 2.46	21.81 ± 2.99	0.777

MMSE: mini mental state examination; BDI-II: Beck Depression Inventory, 2nd edition; *: Post hoc analyses indicated that both values of VO_2max_ are significantly higher in the open- and closed-skill groups than the control group (open-skill vs. control: p = 0.010; closed-skill vs. control: p < 0.001).

### Experimental Procedure

The participants were required to make two visits to the cognitive neurophysiology laboratory. On the first visit the research assistant explained the experimental procedure, and asked the participants to complete a medical history and demographic questionnaire, MMSE, DBI II, a handedness inventory (Chapman and Chapman, 1987), social participation questionnaire (Wu, 2011), and an informed consent form. VO_2max_ was estimated by the Rockport Fitness Walking Test (Kline et al., 1987), in which the participants were required to walk one mile as quickly as possible, during which their heart rate (HR) was continuously recorded using a Polar HR monitor (RX800CX, Finland). Working memory was estimated by the digit span component of the Wechsler-IV Adult intelligence test (Wechsler, 2008). All of the participants’ heights and weights were also measured to calculate their body mass indexes (BMI).

**Figure 1:**
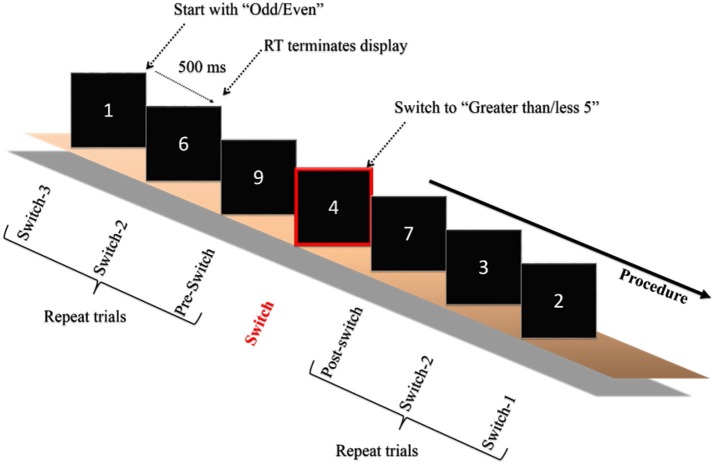
**Schematic of the experimental paradigm**. Only one example of the switch blocks is depicted. Participants began with one of the two tasks (odd/even or greater/less than 5) and were required to switch when a rectangle surrounded the digit. The digit remained on the screen until the participant responded, which initiated a 500 ms interval, after which the next digit was presented. The paradigm was adapted from Friedman et al. (2008).

On the second visit in the same week the experimenter explained the experimental procedure after the participants arrived at the laboratory. The experiment was carried out in an acoustically shielded room with dimmed lights. Initially, the electrocap and electro-oculographic (EOG) electrodes were attached to the subjects’ heads and faces before the test. Each participant was then asked to sit comfortably in front of a 17″ computer monitor, the display of which was driven by an IBM compatible personal computer with a stimulation system (Neuroscan Ltd., EI Paso, TX, USA). The distance between the computer screen and the participant was 75 cm. The task switching paradigm employed in the current study was adapted from one previously used by Friedman et al. (2008) with older adults. The stimulus was a white digit (1–9, excluding 5) presented focally in the center of the screen on a black background. The same digit was never repeated in successive trials and all of the digits were grouped into eight task blocks (blocks 1–2 and 7–8: homogeneous tasks; blocks 3–6: heterogeneous tasks), with a brief rest period in the middle of each block. The homogenous (i.e., non-switch) blocks were composed of 56 trials each. Within the homogenous block, participants only responded if the digit was more or less than 5 (e.g., blocks 1 and 7), or if the digit was odd or even (e.g., blocks 2 and 8). The heterogeneous (i.e., task-switches) blocks were composed of 112 trials, each with 10 switches. Within the heterogeneous block, participants began with one task (e.g., even/odd) and then had to switch to the other (e.g., more/less than 5), which was signaled by a simultaneously presented rectangle drawn around the digit, after a minimum of seven or a maximum of 13 intervening trials (See Figure 1). Participants had to press one of two buttons on a small response box held in both hands as quickly and accurately as possible. The digit was presented on the screen until the participants pressed the response button. The next trial began 500 ms following the RT response. The prompts “more less” or “even odd” appeared simultaneously with and below the digit for all trials, depending upon the side of response that was appropriate to the task. The response hand and homogenous/heterogeneous blocks were counterbalanced across participants. Participants were given the task instructions, and single-task as well as task-switch trials were practiced before the formal test until the participants understood the whole experimental procedure.

### Electrophysiological Recording and Analysis

Electroencephalographic (EEG) activity was recorded from 18 scalp sites (F7, F8, F3, F4, Fz, T3, T4, C3, C4, Cz, T5, T6, P3, P4, Pz, O1, O2, and Oz), using an elastic electrode cap (Quik-Cap, Compumedics Neuroscan, Inc., El Paso, TX, USA) designed for the International 10–20 System for scalp placement. Horizontal and vertical EOG activity for eye movements was monitored bipolarly with ocular electrodes placed on the supero-lateral right canthus and infero-lateral to the left eye. Scalp locations were referred to linked mastoid electrodes, while a ground electrode was placed on the mid-forehead on the Quik-Cap. Interelectrode impedance was below 5 kΩ. EEG and EOG signals were recorded with an A/D rate of 500 Hz/channel, a band-pass filter of 0.1–50 Hz, and a 60 Hz notch filter. These data were continuously written to hard disk for off-line analysis using SCAN4.3 analysis software (Compumedics Neuroscan, Inc., El Paso, TX, USA).

ERP analysis epochs extracted off-line consisted of segments from −200 ms of pre-stimulus activity to 800 ms of post-stimulus activity. ERP averages were computed for each of the digits during the switch blocks. Trials with a response error or EEG artifacts (e.g., VEOG, HEOG, and electromyogram) exceeding peak-to-peak deflections over 100 µV were rejected before averaging.

Since the effects of task switching on the P2 and P3 components in the elderly were clearly visible in frontal-central regions of the scalp in the current study (see also Kieffaber and Hetrick, 2005; Friedman et al., 2008), six electrodes (F3, Fz, F4, C3, Cz, C4) were analyzed in the current work. Two types of ERP variables (i.e., amplitudes and latencies) were measured. P2 and P3 mean amplitudes were calculated for 150–250 ms and 350–600 ms time intervals, respectively. Latencies were measured within the latency window for every participant, determined using the group grand average waveforms, and the results were equivalent for ERP elicited by all conditions and participants.

### Data Processing and Statistical Analysis

Four conditions were subjected to the behavioral and ERP statistical analyses: one (i.e., non-switch trial) during homogeneous task blocks, and three (i.e., pre-switch, switch, and post-switch trials) from switch blocks according to the digit position relative to the switch trial (Friedman et al., 2008). Only the behavioral and ERP data corresponding to correct responses were analyzed. Three types of switch cost were determined by the RTs performance: (1) general-switch cost was determined by subtracting the mean RT between non-switch trials during homogeneous blocks and pre-switch trials during heterogeneous blocks; (2) specific-switch cost was determined by subtracting the mean RT between pre-switch trials and switch trials during heterogeneous blocks; and (3) post-switch cost was determined by subtracting the mean RT between pre-switch trials and post-switch trials during heterogeneous blocks.

The results for the separate behavioral performance (e.g., accuracy rate and RTs) and ERP components (i.e., P2 and P3 latencies and amplitudes) were analyzed using a mixed design, factorial, and repeated-measures analysis of variance (ANOVA). With regard to the behavioral performance of the task switching paradigm, *Group* (open-skill vs. closed-skill vs. control) was the between-subjects factor, and *Condition* (pre-switch, switch, post-switch, and non-switch trial types) was the within-subject factor, with the accuracy rates and mean RTs of accepted trials serving as the dependent variables. For the ERP P2 and P3 measures, *Group* (open-skill vs. closed-skill vs. control) was also the between-subjects factor, and *Condition* (pre-switch, switch, post-switch, and non-switch trial types) and *Electrode* (F3, Fz, F4, C3, Cz, C4) were the within-subjects variables. Where a significant difference occurred, Bonferroni *post hoc* analyses were performed. Significant alpha levels were adjusted with the Greenhouse-Geisser (G-G) epsilon correction whenever a major violation of the sphericity assumption was detected in repeated measures ANOVA, with more than two degrees of freedom (Vasey and Thayer, 1987). The effect size (i.e., partial *η*^2^: $\eta_{p}^{2}$) is also reported to complement the use of significance testing, with the following conventions adopted to determine the magnitude of the mean effect size: <0.08 (small effect size), between 0.08 to 0.14 (medium effect size), and >0.14 (large effect size). Since the correction factor reduces the degrees of freedom of the usual *F*-test, and often results in non-integer values, only the corrected probability values and degrees of freedom are reported. In addition, since the neural processes responsible for switch costs are elicited at the time of target presentation (Kieffaber and Hetrick, 2005), sensitivity indices (general-, specific, and post-switch cost for P2 and P3 latency and amplitude) were also calculated, and the mean amplitudes and latencies of P2 and P3 were submitted to a series of Pearson product-moment correlation analyses with the mean RT switch costs (i.e., general-, specific-, and post-switch costs), respectively. A value of *p* < 0.05 was considered to be significant.

## Results

As shown in Table 1, the three (i.e., open-skill, closed-skill, and control) groups were matched at the group level on age, height, weight, BMI, systolic and diastolic pressure (all *p*s > 0.05). The levels of education, cognitive impairment, social participation, depression, and memory depth (all *p*s > 0.05) also revealed non-significant differences across the three groups. Only the cardiorespiratory fitness showed significant differences among the three groups (*F*_(2,61)_ = 10.63, *p* < 0.001). *Post hoc* comparisons demonstrated that the open- and closed-skill groups had significantly larger maximal oxygen uptake (VO_2max_) than the control group (open-skill vs. control: *p* = 0.001; close-skill vs. control: *p* < 0.001), and the level of cardiorespiratory fitness was matched between the open- and closed-skill groups (*p* = 0.441).

### Behavioral Performance

**Figure 2:**
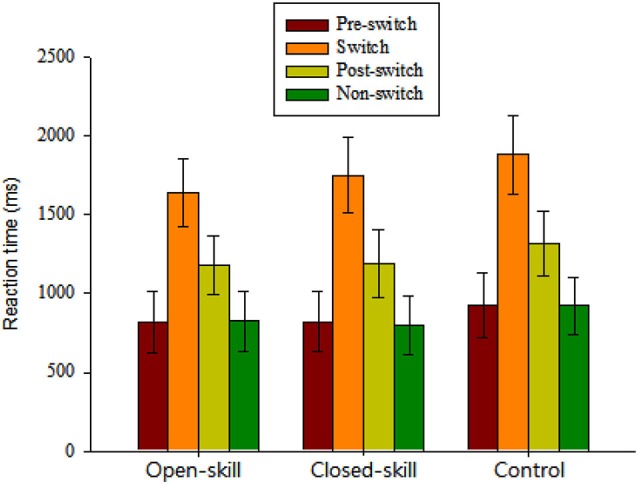
**Grand mean RT data (mean ± SD) for pre-switch, switch, and post-switch trials during the heterogeneous blocks and non-switch trials during homogeneous blocks in the open-skill, closed-skill, and control groups (Note: The control group showed overall slowing relative to the two exercise groups across the four conditions; the open-skill group responded faster in the switch condition than the close-skill or control groups)**.

As seen in Figure 2, the results of repeated-measures ANOVA on the mean accuracy rate revealed a significant main effect of *Condition* (*F*_(3,183)_ = 9.06, *p* < 0.001, $\eta_{p}^{2}$ = 0.13), indicating that a higher percentage of errors were found in the switch (88.0%) and post-switch (87.7%) conditions relative to pre-switch (91.3%) and non-switch (90.8%) ones. Neither significant main effects of *Group* (*F*_(2,61)_ = 0.04, *p* = 0.957) nor significant interactions between *Group* and *Condition* (*F*_(6,183)_ = 2.07, *p* = 0.093) were obtained.

The repeated-measures ANOVA on the grand mean RT data revealed a significant main effect of *Group* (*F*_(2,61)_ = 3.35, *p* = 0.042, $\eta_{p}^{2}$ = 0.10), indicating that the control group (1261.73 ms) showed overall slowing relative to the two exercise groups (open-skill: 1116.21 ms; closed-skill: 1141.6 ms) across the four conditions. The main effect of *Condition* (*F*_(3,183)_ = 1861.58, *p* < 0.001, $\eta_{p}^{2}$ = 0.97) was also significant, with the following gradient: switch (1759.4 ms) > post-switch (1229.64 ms) > pre-switch (855.65 ms) > non-switch (847.68 ms). These main effects were superseded by the *Time* × *Condition* (*F*_(6,183)_ = 4.62, *p* = 0.001, $\eta_{p}^{2}$ = 0.13) interactions. *Post hoc* analyses indicated that the open-skill group responded faster in the switch condition than the closed-skill or control groups.

In terms of RT switch costs, one-way ANOVA showed only a significant main effect of *Group* (*F*_(2,61)_ = 7.30, *p* = 0.001) for the specific-switch cost. *Post hoc* analyses indicated a smaller specific-switch cost in the open-skill group (820.52 ± 112.54 ms) than in the closed-skill (940.30 ± 144.3 ms) or control groups (959.35 ± 124.51 ms).

### Electrophysiological Performance

**Figure 3:**
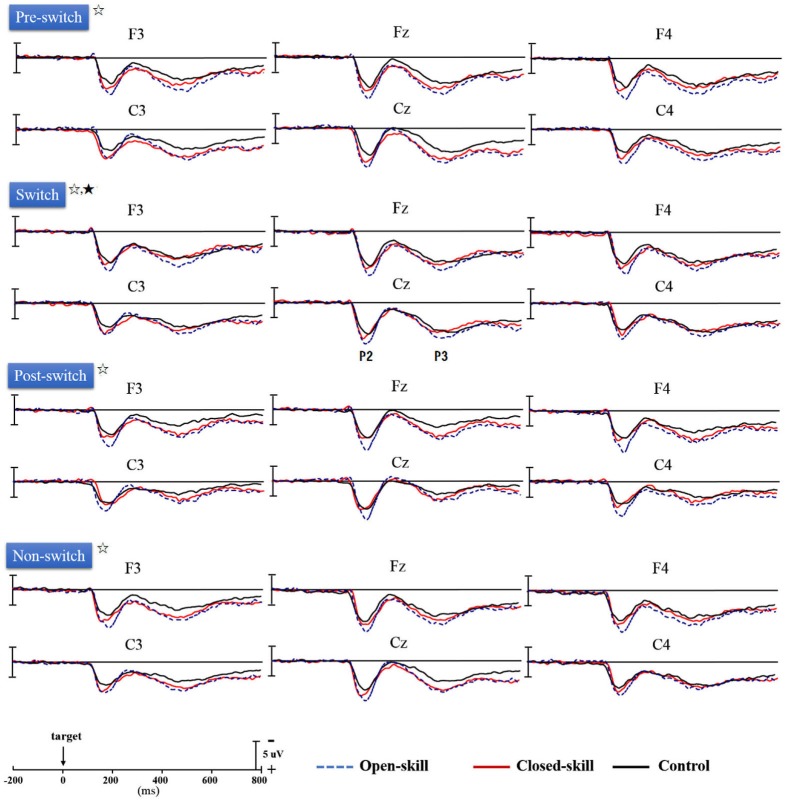
**Grand-average ERPs for four conditions (pre-switch, switch, post-switch, non-switch) at six electrodes (F3, Fz, F4, C3, Cz, C4) in the open-skill, closed-skill, and control groups (☆: open- and closed-skill groups showed significantly larger P2 and P3 amplitudes than the control group.; ★: The open-skill group had a significantly larger P3 amplitude than the closed-skill and control groups)**.

**Figure 4:**
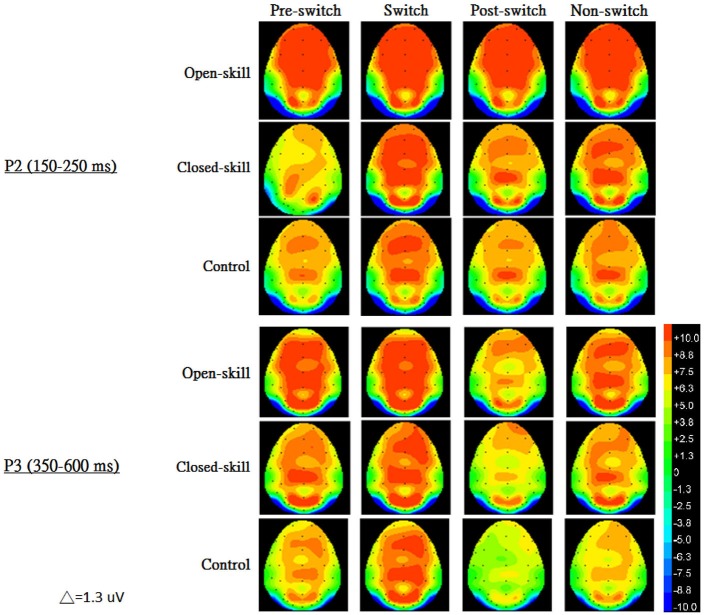
**The topography scalp distribution maps of the stimulus-elicited positivities, P2 and P3 event-related potential components, for four conditions (pre-switch, switch, post-switch, non-switch) in the open-skill, closed-skill, and control groups**.

Figure 3 shows the grand-average ERP waveforms obtained from the six electrodes in the three groups. The topography scalp distribution maps of the stimulus-elicited positivities, P2 and P3 event-related potential components, for four conditions in the three groups are illustrated in Figure 4.

### P2 Latency

As seen in Figure 3, there was only a significant effect of *Electrode* (*F*_(5,305)_ = 4.68, *p* = 0.002, $\eta_{p}^{2}$ = 0.07) on the P2 latency, with the following gradient: Cz (190.42 ms) > C4 (187.62 ms) > C3 (187.34 ms) > F4 (184.39 ms) > Fz (183.64 ms) > F3 (183.51 ms). No significant difference was observed between the three groups in the P2 latency, without any other main effect or interaction.

### P2 Amplitude

As seen from Figure 3, *Group* had significant effects (*F*_(2,61)_ = 12.88, *p* < 0.001, $\eta_{p}^{2}$ = 0.30) on the P2 amplitude, indicating that the open-skill (10.42 ± 2.10 µV) and closed-skill (8.44 ± 2.25 µV) groups showed significantly larger P2 amplitudes than the control (6.92 ± 2.38 µV) group (open-skill vs. control, *p* < 0.001; closed-skill vs. control, *p* = 0.016) across all conditions. There were significant effects of *Condition* (*F*_(3,183)_ = 5.96, *p* = 0.001, $\eta_{p}^{2}$ = 0.09) and *Electrode* (*F*_(5,305)_ = 45.32, *p* < 0.001, $\eta_{p}^{2}$ = 0.43) on the P2 amplitude, with the following gradient: switch (9.21 µV) > non-switch (8.78 µV) > pre-switch (8.49 µV) > post-switch (7.91 µV) in the four conditions, and Fz (9.55 µV) > Cz (9.27 µV) > F4 (8.94 µV) > F3 (8.51 µV) > C4 (8.05 µV) > C3 (7.23 µV). The effects of the interactions of *Group* × *Electrode* (*F*_(10,305)_ = 3.25, *p* = 0.001, $\eta_{p}^{2}$ = 0.10), *Condition* × *Electrode* (*F*_(15,915)_ = 3.52, *p* < 0.001, $\eta_{p}^{2}$ = 0.06), and *Group* × *Condition* × *Electrode* (*F*_(30,915)_ = 1.86, *p* = 0.004, $\eta_{p}^{2}$ = 0.06) on P2 amplitudes were also significant. However, the effect of the interaction of *Group* × *Condition* was not significant.

### P3 Latency

No significant difference was observed between the three groups in the P3 latency, without any other main effect or interaction (see Figure 3).

### P3 Amplitude

As shown in Figure 3, *Group* had significant effects (*F*_(2,61)_ = 8.21, *p* = 0.001, $\eta_{p}^{2}$ = 0.21) on the P3 amplitude, indicating that the open-skill (7.77 ± 2.09 µV) and closed-skill (6.72 ± 2.24 µV) groups showed significantly larger P3 amplitudes than the control (5.00 ± 2.40 µV) group (open-skill vs. control, *p* = 0.001; closed-skill vs. control, *p* = 0.043) across all conditions. There were significant effects of *Condition* (*F*_(3,183)_ = 19.66, *p* < 0.001, $\eta_{p}^{2}$ = 0.24) and *Electrode* (*F*_(5,305)_ = 19.27, *p* < 0.001, $\eta_{p}^{2}$ = 0.24) on the P3 amplitude, with the following gradient: pre-switch (7.28 µV) > switch (7.16 µV) > non-switch (6.40 µV) > post-switch (5.13 µV) in the four conditions, and F4 (7.30 µV) > Fz (6.96 µV) > Cz (6.60 µV) > F3 (6.18 µV) > C3 (6.05 µV) > C4 (5.87 µV). The effects of the interactions of *Group* × *Condition* (*F*_(6,183)_ = 2.37, *p* = 0.031, $\eta_{p}^{2}$ = 0.07), *Group* × *Electrode* (*F*_(10,305)_ = 3.01, *p* = 0.001, $\eta_{p}^{2}$ = 0.09), and *Condition* × *Electrode* (*F*_(15,915)_ = 6.25, *p* < 0.001, $\eta_{p}^{2}$ = 0.09) on P3 amplitudes were also significant. In terms of the significant effect of *Group* × *Condition*, *post hoc* analyses revealed that, compared to the control group, the P3 amplitudes were significantly larger in the two exercise groups in the pre-switch, post-switch, and non-switch conditions; and the open-skill group (8.69 ± 2.99 µV) had a significantly larger P3 amplitude than the closed-skill (6.44 ± 2.78 µV) and control groups (6.35 ± 3.00 µV) (open-skill vs. closed-skill: *p* = 0.043; open-skill vs. control: *p* = 0.035) only in the switch condition.

#### Correlation Analysis

Neither P2 nor P3 performance showed any pattern of significant correlations with behavioral responses in the general-, specific-, and post-switch costs in the three groups, respectively.

## Discussion

The purpose of this study was to explore the exercise-mode-related changes in task-switching performance in the elderly. The results of study confirm that, relative to the control group, the two exercise groups exhibited shorter RTs and larger P2 and P3 amplitudes across all conditions. In addition, the elderly subjects participating in the open-skill exercise showed smaller specific costs, and faster motor RTs and larger P3 amplitudes in the switch condition when compared to the closed-skill or control groups when performing the task-switching paradigm.

With regard to behavioral performance, the RT results on the four conditions indicate that the four trial types required increasing amounts of executive processing when the participants performed the task switching paradigm, consistent with a previous study (Friedman et al., 2008). That is, relative to the pre-switch trials, switch and post-switch trials engendered considerable prolongation of RTs, which produced specific switch and post-switch costs, respectively. The difference in RTs among the three groups was present across the four conditions of the task-switch paradigm, in which the control group showed significantly slower response times when compared to both the open- and closed-skill groups, suggesting that the elderly subjects who regularly participated in physical exercise displayed a generalized reduction in the time efficiency of the central processing of cognitive functions in comparison to the sedentary-behavior group. This is compatible with the findings in Hillman et al. (2006) and Dai et al. (2013) that elderly people with higher levels of physical activity or regular participation in open- or closed-skill exercise showed shorter RTs than those with less physical activity or irregular exercise. However, no differences in accuracy rates among the three groups were found in this study, and this supports the view that the RT differences across all conditions could be attributed to the differences in processing time between the control group and the two exercise groups, demonstrating that the generalized reduction in the time efficiency of the central processing of cognitive functions could be regarded as a general advantage of regular participation in sports and other forms of exercise over a sedentary lifestyle (Di Russo et al., 2010; Tsai et al., 2014a, 2015).

On the other hand, the open-skill group showed faster RTs in the switch condition than the closed-skill or control groups. Given that participating in the open-skill exercise modes could facilitate differential advanced preparation for an upcoming switch (Di Russo et al., 2010), these findings indicate that the elderly people engaging such an exercise mode could become better at switching from one task to another, as this constantly happens in an open-skill exercise environment. The open-skill group also had a lower specific-switch cost than the closed-skill and control groups, in contrast to the results in Dai et al. (2013), which reported that the facilitative effect of open-skill exercise was not observed in local switch costs (i.e., the RT differences between the switch and non-switch trials in the heterogeneous condition). There are several plausible mechanisms for the contrasting results. First, the switch predictability in switch-related processing was different in the task-switching paradigms used in the current and earlier works. Dai et al. (2013) adopted a series of pair-trials (e.g., AABBAABB) in their cognitive task, which made a greater demand on working memory, because the participants needed to remember where they were in the sequence. In the current study, one task (e.g., even/odd) switches to the other (e.g., more/less than 5) after a long repetition of sequences (i.e., a minimum of seven or a maximum of 13 intervening trials), and this design could produce a great deal of interference from the prior task set on the switch trial. Moreover, the infrequent occurrence of switch trials (less than 10%) in the present study meant that it was difficult for the participants to predict when a switch would occur, and thus they were likely to adopt a strategy of keeping the current task set in their working memory, and reconfigured this to suit a new task set only when a change in task was signaled by the cue (Monsell and Mizon, 2006; Friedman et al., 2008). Further, given the lack of a cue-and-target preparatory interval in the task-switching paradigm in the present study, the participants did not have enough time to reallocate attention to the new task set according to the cue preceding the target, nor update the procedural rules by retrieving them from memory. Since open-skill exercise could favor response flexibility and foster greater stability of motor responses, and the open-skill exercise environment is unpredictable and always changing (Di Russo et al., 2010), the elderly subjects who regularly participated in such an exercise mode could see greater cognitive flexibility when switching from pre-switch to switch trials during the heterogeneous conditions, as designed in the current study.

In addition, no difference in the general-switch cost was found in the current study, suggesting that the process of selecting between and coordinating the two competing tasks appeared to be comparable among the three groups (Friedman et al., 2008). However, the facilitative effect of open-skill exercise in the elderly was observed with regard to the global switch cost (i.e., the RT differences between the homogeneous and heterogeneous conditions) in a previous study (Dai et al., 2013). As noted earlier, these discrepancies may result from procedural differences in the task-switching paradigm. Since Dai et al. (2013) adopted an alternating runs paradigm with a lack of switch cues, the participants had to keep track of which task rules were operative and the sequence of the trials, which would produce higher working memory loads. In contrast, when the participants performed the task-switching paradigm in the current study, a cue carried task-relevant information to remind them of the response key assignments on each trial. Accordingly, the lower working memory demands could, at least in part, have led to the similar general-switch costs found in the present study (Friedman et al., 2008).

Event-related potential P2 is associated with a unique component of switch cost (Kieffaber and Hetrick, 2005). In the present study, the P2 latencies were not significantly different between the three groups. However, there was a significant *Group* effect on the P2 amplitude, indicating that two exercise groups showed significantly larger P2 amplitudes across all conditions relative to the control group. Previous studies have demonstrated that the elderly need to meet more processing demands during maintenance and retrieval of two task sets concurrently held in working memory when performing a task-switching paradigm task (DiGirolamo et al., 2001; Goffaux et al., 2006). Since P2 amplitude is sensitive to task switching when a shift of target modality is involved (Kieffaber and Hetrick, 2005; Adrover-Roig and Barceló, 2010), the findings of the present study thus lend support to the hypothesis that regularly participating in the physical exercise (e.g., open- and closed-skill modes) could facilitate better cognitive control with regard to task-set activation (i.e., inhibitory control of responses for a previously performed task) among elderly subjects (Cepeda et al., 2001; Periáñez and Barceló, 2009), and/or enhanced cue-task retrieval processes (West and Travers, 2008). Although none of the earlier studies tried to explore the relationship between exercise and cognition with regard to the effects of this on the P2 components when elderly subjects performed a task-switching paradigm task, one previous study did find a positive association between physical activity and error-related negativity in elderly individuals, reflecting a greater efficiency in dealing with the conflicts arising during trials among those subjects who engaged in more physical activity (Themanson et al., 2006). It is worth pointing out that P2 component is associated with switch cost (Kieffaber and Hetrick, 2005), and thus the two exercise groups which showed larger P2 amplitudes should exhibit better performance with regard to these costs. However, only the open-skill group had smaller specific-switch costs when compared to the closed-skill and control groups. Indeed, previous studies found that the magnitude of switch costs does not depend on the amount of physical activity in the elderly (Hillman et al., 2006; Themanson et al., 2006). In addition, Themanson et al. (2006) found that sedentary elderly adults who engaged in erobic exercise (a closed-skill exercise mode) for 6 months showed a significantly greater reduction in the magnitude of the switch cost when compared to those in the strength and flexibility exercise (also a closed-skill exercise mode) group. Given the results of the current and previous studies, cardiorespiratory fitness seems to be the potential facilitative factor with regard to the P2 amplitude in the elderly. However, only the elderly who engaged in open-skill exercise and also had a higher cardiorespiratory capacity exhibited the beneficial effect on specific-switch cost. In addition, the switch sensitivities of the P2 and P3 components were not related to the RTs in the general-, specific-, and post-switch costs in the current study, indicating that the stimulus-dependent neural processes may not contribute to RT switch costs in the current task-switching paradigm, and the elderly with higher cardiorespiratory fitness via different exercise modes could see distinctive behavioral and electrophysiological effects.

The P3 latencies were not significantly different among the three groups, suggesting that all participants showed similar perceptual/central processing (Polich, 1997) when performing the task-switch paradigm task in the current study. The main *Group, Condition*, and *Group × Condition* effects on P3 amplitude were revealed in the present study. The P3 amplitude observed in the current study reflects task-set updating processes and attentional allocation to the stimuli in the service of updating the memory (Polich, 1997; Friedman et al., 2008). There were significant differences in the P3 amplitudes among the four conditions in the current task-switching paradigm for the elderly participants, demonstrating that the frontal-central networks responsible for task-set updating processes and attentional allocation were not compromised in the elderly subjects in the present study when performing the cognitive task. Additionally, this finding also suggests that the elderly participants’ reconfiguration of response mapping specific to different conditions was intact, which could appropriately reflect the neural mechanisms at work in processing the current task-switching paradigm. In terms of between-group comparison, the two exercise groups showed larger P3 amplitudes across all conditions relative to the control group, similar to the finding in Hillman et al. (2006) of a larger P3 amplitude for the active rather than for the sedentary elderly when performing the task switching paradigm. Given the lack of preparatory interval and the lower infrequency of switches in the cognitive task, the participants must allocate their attentional resources to predict when a switch would occur and to prepare a new task set before cue occurrence. Since elderly people have been found to have difficulties in reallocating attentional resources (Friedman et al., 2001), and to suffer from age-related disturbances in the task-set updating process (West and Moore, 2005), the results of the current study indicate that regularly participating in physical exercise seems to facilitate the attentional set that makes it possible to better evaluate the stimulus in either of the two tasks.

The open-skill group showed a significantly larger P3 amplitude in the switch condition when performing the task-switching paradigm compared to the closed-skill and control groups. Since the P3 amplitude in such a condition reflects the collection of processes subsumed under the construct of the task-set reconfiguration (Karayanadis et al., 2003; Kieffaber and Hetrick, 2005; Nicholson et al., 2005), and cognitive processing plasticity can be modulated by the execution of the open-skill exercises (Fontani et al., 1999; Iwadate et al., 2005), the current study seems to demonstrate that participating in the open-skill exercise mode could enhance these cognitive processes in the elderly subjects. However, the task-set updating processes in the homogeneous task, pre-switch condition, and post-switch condition were not be affected by the different types of exercise. Additionally, the larger P3 amplitude of the open-skill group only appeared in the switch condition, implying that the enhanced P3 amplitude was selective for cognitive processing related to tasks requiring stimulus perception and identification, task-set updating, response conflict detection and monitoring processing (Friedman et al., 2008). However, this finding for P3 amplitude differed to that in a previous study (Dai et al., 2013), in which the closed-skill and open-skill groups showed similar electrophysiological performances in the P3 component (e.g., P3 amplitude and P3 latency) in the homogenous and heterogeneous blocks. The most likely explanation for the contrasting results might be the elderly subjects’ exercise experience (3 months in Dai et al.’s (2013) study vs. 24 months in the current study), and the different characteristics of the task-switch paradigm, as mentioned above. Since attentional allocation and cognitive processing are more susceptible to the effects of interceptive sports (e.g., badminton and table tennis) than static ones (e.g., swimming and jogging) (Voss et al., 2010), and cognitive adaptations (e.g., strengthened synaptic neurotransmission and increased neurogenesis) are available in complex environments that engage rich cognitive loadings (van Praag et al., 2000; Artola et al., 2006; Gajewski et al., 2010), we assumed that the elderly subjects participating in the open-skill exercise for longer periods would see greater behavioral and neurobiological consequences with regard to specific switching aspects of the executive functions (van Praag et al., 2000; Dai et al., 2013).

Although we rigorously controlled some important factors which could mediate the exercise-cognition association (e.g., blood pressure, depression and social stimulation, Miller et al., 2012), there are still some potential limitations in the current study. First, the elderly subjects with higher cardiorespiratory fitness (i.e., open- and closed-skill groups) showed better behavioral and electrophysiological performances when performing a cognitive task involving executive control compared to those with lower cardiorespiratory fitness (i.e., the control group). The results conform to the trend seen in a large number of previous studies (Hillman et al., 2006; Themanson et al., 2006; Netz et al., 2011; Frederiksen et al., 2014), showing that a general reduction in cardiorespiratory fitness and lower physical activity levels may contribute to worse executive control functioning. However, when the elderly subjects with higher cardiorespiratory fitness were categorized into open- or closed-skill exercise mode groups in the present study, we found more detailed differences in the behavioral and electrophysiological performances between the two elderly groups, as mentioned above. However, previous studies demonstrated that cardiorespiratory fitness is the main factor that affects cognitive processing (Stroth et al., 2009; Tsai et al., 2014b). It remains an open question as to whether elderly subjects participating in the open-skill exercise mode and with abundant exercise experience, but without higher cardiorespiratory fitness, could still exhibit different cognitive performances on specific switch aspects of executive functions in the task-switching paradigm. Future research efforts should thus address this issue. Secondly, the present experiment was a cross-sectional study, and thus the elderly subjects participating in the open-skill exercise mode might inherently have had better executive functioning (e.g., task switching) compared to their counterparts participating in the closed-skill exercise mode, and this may have induced them to adopt this kind of exercise, and thus also show better behavioral and electrophysiological performances in the switch condition when performing the task-switching paradigm. Future studies could thus examine the contributions of different exercise-mode mechanisms responsible for the specific types of executive-control functioning via longitudinal experiments regarding specific exercise-mode interventions, in which elderly subjects without any exercise experience are randomly assigned to different exercise modes (Snowden et al., 2011).

A healthy lifestyle is well known to protect against the development of numerous medical disorders. Indeed, the elderly subjects who regularly participated in open- or closed-skill exercise exhibited better behavioral and electrophysiological performances when performing the task-switching paradigm in the current study. Additionally, given that participating in open-skill exercise stimulates specific types of executive-control functioning, open-skill exercise (e.g., table tennis or badminton) could be an effective physical activity mode that can help elderly people to better deal with two competing tasks and task-set/memory updating processing, especially in the switch condition.

## Conflict of interest statement

The authors declare that the research was conducted in the absence of any commercial or financial relationships that could be construed as a potential conflict of interest.
